# Uterine Hemorrhage

**Published:** 1876-04

**Authors:** 


UTERINE HEMORRHAGE.—*The menopause.*—Dr. Hanks is very decidedly of the opinion that the *physiological* flooding at the change of life is not great, and that when it is profuse it is invariably due to some pathological cause. Cancer very frequently develops at this period, and, as the hemorrhage really occasioned by it is supposed to be simply one of the normal phenomena attending the menopause, the presence of malignant disease is often not suspected until it has made alarming progress.

*Fibroids.*—Dr. Hanks has obtained very fair results in the treatment of these growths by the internal and hypodermic use of ergot, combined with tonics; and he detailed the history of a large subperitoneal one, which was diminished one-fourth in size in two months by this means.

*Carcinoma.*—Here he recommended the application of sesquichloride of iron twice a week, and in some cases simply alum; but made no mention of the use of the actual cautery or galvano-cautery, which have proved so signally effective in arresting and preventing hemorrhage from cancer.

*Retroversion.*—This is apt to be associated with, and, indeed, is really caused by congestion and endometritis. In the treatment of the malposition, Dr. Hanks, after restoring the organ, makes use of the cotton and glycerine packing which Professor Thomas almost invariably employs before inserting a pessary.

*Fungoid excrescences on the mucous membrane of the uterus.* The diagnosis of this condition of the endometrium is to be primarily arrived at by a process of exclusion, and will, of course, be confirmed when any of the little congeries of vessels in tufts are detached. The treatment by means of Thomas' flexible wire curette is most safe and effective.

*Ulceration and erosion* (chronic granular os) are generally accompanied by changes in the mucous membrane of the uterus, and by displacements, and these must be corrected, as well as the ulceration. The best application in the treatment of the latter is strong nitric acid, frequently repeated, and followed by the use of water. Iodoform and the sesquichloride of iron Dr. Hanks has also found useful; and as the case progresses towards recovery he employs milder agents. He is very strongly opposed to the use of the nitrate of silver, which he believes occasions contraction of the os and is a very frequent cause of dysmenorrhœa and sterility.

Dr. Hanks added greatly to the value of his paper by the recital of cases illustrative of the various conditions giving rise to uterine hemorrhage taken from his own experience, which has been extensive in connection with Prof. Thomas' clinic and the gynœcological department of the Demilt Dispensary, as well as in private practice.

The latter part of it was devoted to hemorrhages incident to the puerperal state.

*Retained placenta.*—He recommends the bi-manual operation for the removal of the placenta, and has also found the hypodermic use of ergot of great service. Where there is much exhaustion, he is in the habit of injecting forty minims of brandy with twenty minims of the fluid extract of ergot, and almost invariably with the best results.

*Placenta prœvia.*—In this condition we are first to endeavor to cause the active contraction of the uterus, the fœtus serving as a tampon in the os ; and, failing in this, we must dilate the os, and deliver as rapidly as possible.

*Post-partum hemorrhage from inertia.*—Here his great reliance is the hypodermic injection of ergot, and compression over the uterus with the hand, which should be kept up by the nurse or other attendants in some cases for twelve or even twenty-four hours.—*Philadelphia Med. Times.*

